# Referral Patterns in Oral Medicine: A Retrospective Analysis of an Oral Medicine University Center in Southern Italy

**DOI:** 10.3390/ijerph182212161

**Published:** 2021-11-19

**Authors:** Noemi Coppola, Stefania Baldares, Andrea Blasi, Rosaria Bucci, Gianrico Spagnuolo, Michele Davide Mignogna, Stefania Leuci

**Affiliations:** 1 Oral Medicine Unit, Department of Neuroscience, Reproductive and Odontostomatological Sciences, University of Naples Federico II, 80131 Naples, Italy; noemi.coppola@unina.it (N.C.); dott.baldares@tiscali.it (S.B.); gspagnuo@unina.it (G.S.); mignogna@unina.it (M.D.M.); stefania.leuci@unina.it (S.L.); 2 Department of Neuroscience, Reproductive and Odontostomatological Sciences, Section of Orthodontics and Temporomandibular Disorders, University of Naples Federico II, 80131 Naples, Italy; rosaria.bucci@unina.it; 3 Institute of Dentistry, I. M. Sechenov First Moscow State Medical University, 119435 Moscow, Russia

**Keywords:** oral medicine, oral diseases, referral, quality of referral, consultation, primary healthcare, dentistry

## Abstract

Referral of a patient from one healthcare provider to another is an important part of the medical practice. The aim of this study was to analyze the referral process to the Oral Medicine Unit in a university-based tertiary center in Southern Italy. A chart review of new referrals to the Oral Medicine Unit during a 24-month period was conducted. The following data were recorded: demographic characteristics, medical history, number of physicians seen prior to Oral Medicine assessment, referral source, diagnostic procedures ordered by referrals, reason for referral, site of lesion/condition, final diagnosis. Then, the rates of correct identification for health-care professionals and the appropriateness of the reference diagnosis based on the disease were calculated with descriptive statistic indicators. There were 583 new first consultations. A total of 62.9% of patients were referred by general dental practitioners, 27.4% by physicians, and 9.7% did not have a referral. The most common diseases for referral were immune-mediated diseases (39.6%) and oro-facial pain disorders (25.2%). Only 28.5% of patients had a correct provisional diagnosis. The results of this study show the need to implement curricula in the field of oral medicine among dentistry and medical students, and to support the continuing education among healthcare providers to reduce diagnostic delay for oral diseases.

## 1. Introduction

Referral is an integral part and a critical component of the current medical practice, defined as the taking over care from one healthcare provider to another [1]. The referring physician sends the patient to the specialist physician for evaluation and management of a specific clinical problem [2]. If practised efficiently, it can contribute to high standards of care by improving patient outcomes and decreasing costs through optimal use of medical services. High-quality referral and consultation processes require good communication between healthcare providers: this may lead to reduced delays in the diagnostic care pathway, high-quality continuity of care and improved patient’s satisfaction [3]. The interaction between general medical practitioner (GMP) and specialist physician is based on the recommendation letters where there is the patient’s clinical history, the description of symptoms and a provisional diagnostic hypothesis [4]. In medicine, the analysis of referral patterns is a relevant and current issue as it is closely connected to the quality of healthcare and its costs [5]. To provide quality healthcare by ensuring that the patient receives proper diagnosis and treatment in a timely manner, the referral must have requirements of necessity, adequacy, timeliness and must be well communicated in the letters of recommendation [6]. Furthermore, the quality of the referral process assumes even greater importance in the current historical moment as we are witnessing, on the one hand, an increase in the prevalence of patients that are in chronic maintenance for multiple pathologies (oncological, immunological, neurological, etc.) and, on the other hand, the scarcity of resources to satisfy the demand for specialized care [7]. Referral patterns, therefore, have significant implications in the field of health policies (workforce planning, hospital, and academic funding policy) [8]. Access to oral medicine (OM) is generally based on referrals. OM is defined as “The discipline of dentistry concerned with the oral healthcare of medically complex patients including the diagnosis and management of medical conditions that affect the oral and maxillofacial region” [9]. Scopes of OM are not unique worldwide; in fact, there is no international consensus on the definition and field of action of OM [10]. For example, in Europe, OM is recognised as a specialty only in Croatia, Israel and the United Kingdom. In some European countries, it is an integrated course of study in other dental specialties; in other countries, however, such as in Italy, Spain and Sweden, training in OM is a separate course from other recognized dental disciplines [10]. There is therefore no single training course for OM, which unfortunately is completely lacking in some countries. Despite the great variability depending on the country to which reference is made, OM specialists (OMs) care for patients with a wide range of conditions, including mucosal diseases, oral manifestations of systemic diseases, oral drug-related complications, salivary gland disorders, orofacial pain and temporomandibular conditions [11]. OM inherently has strong relationships with various medical specialities, particularly dermatology, immunology and infectious diseases, pathology, imaging, neurology, oncology, ear-nose-throat specialist (ENT), paediatrics, psychiatry, psychology and rheumatology [12]. Thus, OM is placed at the interface between medicine and dentistry and should ideally serve as a model for interdisciplinary collaboration. According to “Oral Medicine referral guidelines in UK”, the referral letter should include the urgency of the referral, a detailed history of the patient’s complaint, details about prior investigations or treatment performed and a provisional diagnosis, using a red flag for suspicious lesions [13]. Moreover, using high-quality photos may improve the referral process [13]. Retrospective studies on OM referral patterns have been conducted in various countries (Ireland, United States, Australia) [14,15,16]. However, at present, similar data concerning Italy are not available in the literature. Our study aims to describe the different aspects of the referral process to the Oral Medicine Unit (OM-U) in a university-based tertiary referral center located in Southern Italy. In particular, we analyzed source, reason, pertinence and accuracy of the referral diagnosis in the recommendation letters, with the aim of providing a detailed and current picture of services provided in our university and of their use by the dental and medical community in general.

## 2. Materials and Methods

This descriptive study was conducted at the OM-U of the University of Naples Federico II, a specialty referral clinic (Naples, Campania, Italy). This clinic is one of the two referral centers for OM in this region and serves a population of about 5,600,000 (2021). The majority of patients comes from local area; however, some patients from other regions come to this center. We reviewed retrospectively all the new patient’ charts, referred from 1 March 2019 to 31 December 2020. The second year of patient enrollment was marked by the COVID pandemic; therefore, the main enrollment period can be considered until February 2020. Patients who were referred for suspicious oral medicine-related illnesses were included. Patients for whom it was not possible to collect data in detail and for whom a final diagnosis was not indicated were excluded. Each patient underwent a complete clinical interview and examination performed by an OMs.

### 2.1. Data Collection

Data were extracted and recorded from an electronic database by a single researcher. The following variables were identified: age, gender, smoker status (light smokers: <5 cigarettes daily; moderate smokers: 5–10 cigarettes daily; heavy smokers: 15 or more cigarettes daily), alcohol consumption (light drinker: one unit of alcohol daily; moderate drinker: two units/daily; heavy drinker: three or more units/daily), medical history, current drugs, number of physicians seen prior to OM assessment, diagnostic procedures ordered by referrals, referral source, reason for referral, site of lesion/condition, time elapsed between referral and visit to OM-U, final diagnosis by the OMs in according with the International Statistical Classification of Diseases and Related Health Problems (International Classification of Diseases, 11th edition [ICD-11] codes) [17]. Then, the final diagnosis was analyzed by a second researcher in order to categorize into general diagnostic categories proposed by Villa et al.: (1) immune-mediated mucosal conditions, (2) orofacial pain (OFP) disorders, (3) benign tumors or neoplasms, (4) dysplasia and cancerous conditions, (5) reactive keratosis, (6) salivary gland disorders, (7) infections, (8) osteonecrosis of the jaw and (9) other mucosal/gingival and bone conditions [15].

### 2.2. Statistical Analysis

For continuous variables (age, n. of physicians seen prior to OM assessment and time elapsed between referral and visit to OM-U) mean and standard deviation we calculated, whereas non continuous variables (gender, smoking status, alcohol consumption, referral source, diagnostic procedures, reasons for referral, site of the lesion, accuracy of initial diagnosis and category of final diagnosis) were reported as frequencies and percentages. A statistical software (IBM SPSS Statistics v.25, IBM Inc., Armonk, NY, USA) was used for calculation.

## 3. Results

### 3.1. Patient Demographic Characteristics

The demographic characteristics of patients are show in Table 1. During the study period, a total of 583 patients were seen for the first OM consultation. The average age was 56.6 ± 16.2; 218 (37.4%) were males and 365 (62.6%) females. Almost all patients were of Caucasian ethnicity with only 2 from different ethnic groups.

### 3.2. Referral Source

**Figure 1:**
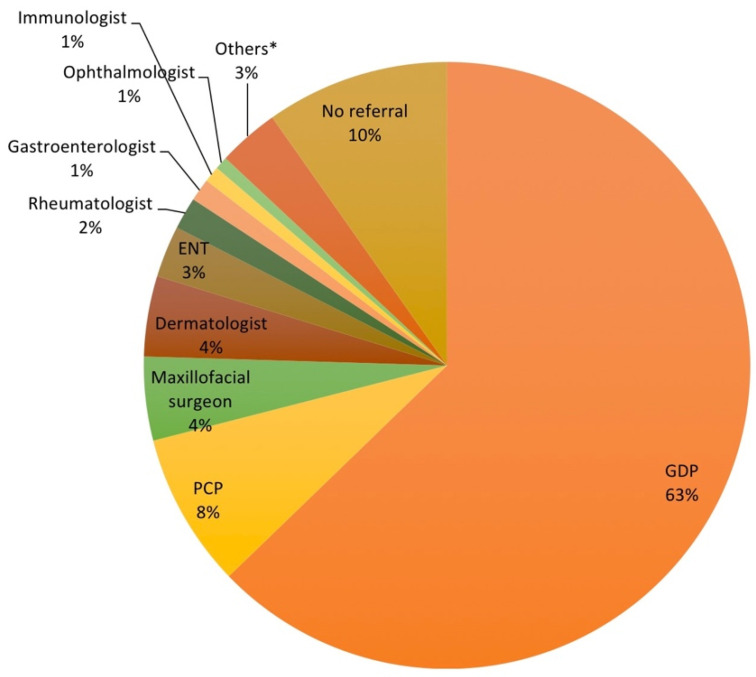
Source of referral. * Others: Oncologist, Pathologist, Plastic surgeon, Neuropsychiatric, Pediatrician, Breast specialist, Allergist, Cardiologist, Hematologist, Gynecologist, Internist, Neurologist. ENT: ear-nose-throat specialist; PCP: primary care physician; GDP: general dental practitioner.

A total of 1163 consultations were performed prior to referral to our university clinic: 439 patients (75.4%) have consulted one or two doctors, 51 (8.8%) consulted three and 72 (12.4%) consulted more than three. Figure 1 shows in detail the sources of the referral. Of the total sample, 366 patients (62.9%) were referred by general dental practitioners (GDPs), 160 (27.4%) by physicians and 57 (9.7%) did not have a referral. From physicians, 48 (8.2% of total referrals) were primary care physicians (PCPs), 26 (4.5%) maxillofacial surgeons, 25 (4.3%) dermatologists and 16 (2.7%) ENT. 

### 3.3. Diagnostic Procedures Ordered by Referral

Before the OM visit, 229 patients (39.3%) underwent diagnostic procedures, the most common of which were: routine blood test (13.7%), biopsy (9.6%) and panoramic radiograph (orthopantomogram-OPG) (9.1%). The other diagnostic tests ordered by referrals are presented in Table 2. Some patients have performed more than one diagnostic test. On the basis of the final diagnosis the appropriateness of the required tests was 41.9% (96/229). The condition for which more incongruous diagnostic tools were ordered was burning mouth syndrome (BMS).

### 3.4. Reasons for Referral

Table 3 shows in details the reason for referral in according to final diagnosis either clinically or histopathologically by OMs. The most common reason for referral was immune-mediated mucosal conditions diagnosed in 231 patients (39.6%) followed by OFP disorders (147/583; 25.2%) and dysplasia and cancerous conditions (81/583; 13.9%). From immune-mediated mucosal disease, the most frequent diagnosis was oral lichen planus (OLP) (164/583; 28.1% of total diagnosis), whereas, from the OFP group, BMS (111/583; 19%) was the main diagnosis, and from dysplasia and cancerous conditions the most common diagnosis was leukoplakia (51/583; 8.8%).

### 3.5. Accuracy of Referrals

**Figure 2:**
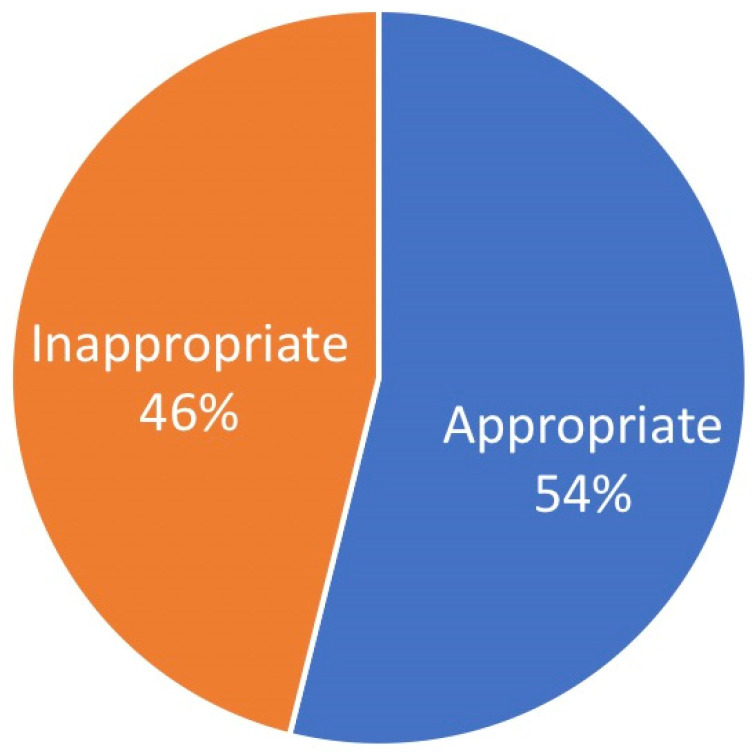
Appropriateness of referral diagnosis.

Only approximately half of the patients was referred with a provisional diagnosis (308 of 583; 52.9%) and, of these, 63.9% (166 of 308) of the initial diagnoses were confirmed (Figure 2).

**Figure 3:**
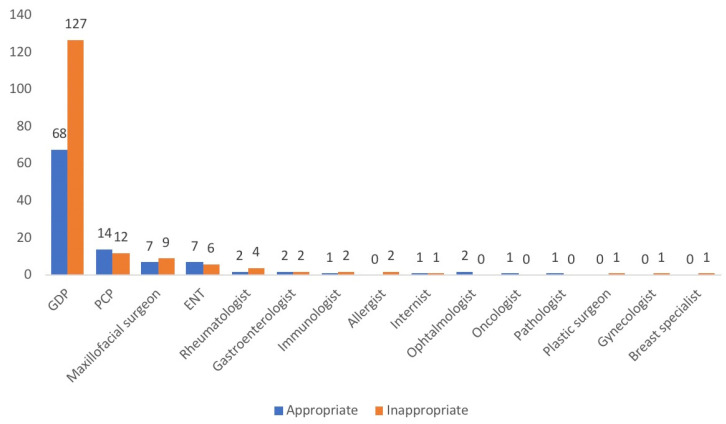
Appropriateness of the reference diagnosis based on the specialty of the referring provider.

The rates of correct identification of the three most frequent conditions, OLP, BMS and leukoplakia, were the following. On a total of 164 cases of OLP, only 97 had a provisional diagnosis and, of these, only 56 (57.7%) were determined to be correct. A total of 51 out of 111 BMS patients were referred with initial diagnosis and 21 (41.2%) were appropriate. From 51 patients with a diagnosis of leukoplakia, 30 had a diagnosis from the referring provider and only 2 (6.7%) were consistent with final diagnosis. Osteonecrosis of the jaws, pemphigus and pemphigoid, aphtosis were the conditions with the highest degree of diagnostic accuracy (91.7%, 66.7%, 66.7% and 58.3%, respectively). The rates of correct identification for health-care professionals (HCP) were the following: GDPs (68/195; 34.9%), PCPs (14/26; 53.8%), maxillofacial surgeon (7/16; 43.7%), ENT (7/13; 53.6%). The diagnostic accuracy of the other physicians is shown in Figure 3.

## 4. Discussion

This single-center study examines the referral pattern related to the OM-U of a University-based hospital in Naples (Italy). In our cohort and as previously reported, the majority of patients were in the fifth decade of life with a female prevalence [15,18]. These data suggest that, to date, OM services are aimed at an adult population and the aging of the general population will increase the demand for assistance in this field [19]. Therefore, with increasing requests, the referral process to the OMs must be appropriate and timely to improve access to healthcare, coordination of care and not waste public health resources. About 75% of patients were visited by at least one doctor before being referred; on average, the number of medical consultations seen is two. The most common sources of referral were GDPs, PCPs, maxillofacial surgeons and dermatologists in line with data from Friesen et al., and Farah et al. [16,18]. Only 27% came from physicians; this percentage is in line with the studies conducted in Australia and in Canada, but in disagreement with Villa et al., where 2/3 of patients were referred by physicians [15,16,18]. A recent systematic review on knowledge, attitude and practice among healthcare providers in oral cancer awareness shows that most of dentists usually referred patients to specialists, instead a minority of medical doctors preferred to refer to specialists, which was rarely an OMs [20]. On the one hand, this can be explained with the different levels of cooperation between dentists and physicians in the healthcare system of various countries, as is also supported by Farah et al. [16]. On the other hand, the low percentage of patients referred by medical doctors could indicate that there is no information among the medical community about the services offered by OM. Moreover, is widely demonstrated that most medical providers consider inadequate the education regarding oral health issues, and they reported that they rarely examine the oral cavity [21,22]. Consequently, the reduced number of referrals by physicians could also be attributed to the non-recognition of oral pathologies due to the lack of oral inspection. Therefore, there are two important needs to improve the management of OM patients. First, it is imperative to raise awareness of the support that OM can provide in the healthcare system. Second, it is also important to encourage continuing training courses in oral medicine for physicians. Although each patient consulted two doctors on average before OM consultation, only half of the patients had a provisional diagnosis and, on a total sample, only 28.5% of patients had an initial diagnosis which was then clinically or histologically confirmed by OMs. GDPs, PCPs and ENT had a higher degree of diagnostic appropriateness among HCPs without a statistically significant difference between dentists and physicians, according to Friesen et al., and Sardella et al. [18,23]. The low rate of diagnostic accuracy makes early diagnosis difficult and highlights that dentists and physicians are not confident with oral diagnosis, and are unable to perform the proper management of clinical patients. This confirms that oral medicine is a specialty which is still finding its place in absence of an international consensus. Whereas in the past years OM have been placed between medicine and dentistry, to date, it is defined as a branch of dentistry even in the absence of shared legislation in the various countries [10,24]. The great diversity both from an educational and clinical point of view worldwide means that there are few healthcare professionals able to satisfy the request for assistance from patients. In particular, to regard the diagnostic procedures, in our study emerged that before the referral to OMs the referring provider ordered or performed diagnostic tool only in 40% of patients. In Italy, diagnostic procedures are provided by the national health system and the choice of resorting to private facilities to carry out medical consultations and assessments depends solely on the will of the patient. Routine blood test followed by biopsy and OPG was the most diagnostic procedure for the assessment of the disease. Compared with the data in the literature, there is a higher percentage of blood examinations and a lower percentage of biopsies performed by other medical professionals, but the biopsy appropriateness rate was higher than in other studies (75%). In fact, in the study by Villa et al., 17.1% of patients had undergone biopsy before the visit with OMs, but more than 1/3 of the biopsies performed were deemed inadequate [15]. Additionally, in our cohort, many diagnostic tests were not appropriate for the patient’s condition, mostly for BMS patients, such as lingual swab, biopsy, and esophago-gastro-duodenoscopy, according to the International Classification of Orofacial Pain [25]. These data are in line with a recent study by Freilich et al., who reported that many BMS patients, despite requiring medical attention, have undergone unnecessary tests and have not been diagnosed or misdiagnosed [26]. Among oral diseases, certainly, BMS is still a challenge for clinicians and for patients [27,28], but any diagnosis in the field of OM is frequently complex. In our study, patients were referred for very heterogeneous diseases, OLP and BMS were the most frequent of them. OFP disorders were the second reasons for referral in line with the increase in the prevalence of chronic pain worldwide, the most common reasons why people seek medical attention [29,30,31]. Although the most frequent pathologies were OLP, BMS and leukolpakia, the most easily diagnosed were osteonecrosis of the jaw bones and autoimmune bullous diseases. These data were in contrast to what emerges from the study by Sardella et al. [23]. In the case of osteonecrosis, the high diagnostic accuracy may be due to the exposure of necrotic bone, pathognomonic feature of this pathology and easy sign to detect. On the other hand, the presence of a highly sensitive and specific test, such as the ELISA test for the detection of anti-desmoglein-1, anti-desmoglein-3, anti-BP180 and anti-BP230 antibodies, may have contributed to the diagnostic precision towards autoimmune bullous diseases. Despite the high percentage of patients with oral cancer risk factors in our cohort, there were relatively few diagnoses of cancerous lesions. Finally, a small percentage of referrals (2.6%) was for dental pathology and paraphysiologic conditions (no evident mucosal pathology, torus mandibularis, tongue ptosis).

As stated above, a referral letter should also include photographs. In our cohort no case was provided with photographic documentation. Several authors suggested that clinical images can be useful for an initial consultation and prioritize [32,33,34,35].

Our study shows that patients with oral diseases are often seen by multiple clinicians before being evaluated by an OMs and may be subjected to unnecessary and often inappropriate diagnostic tests, procedures and therapies. This results in diagnostic delay and delay in the referral to OM-U could worsen the prognosis for many conditions or at least affect the patients’ oral health-related Quality of Life. The high percentage of patients without diagnosis and provisional misdiagnosis indicates the absence of expert figures in OM among health professionals. Furthermore, the high percentage of referrals from dentists indicates that they are the health professionals who are responsible for intercepting the first signs and symptoms of oral diseases. Therefore, to achieve the goal of reducing diagnostic delay and improving healthcare for patients with oral diseases, it is necessary to implement the pre- and post-graduate training education of the dental classes. Only by investing in a broader cultural path can a turning point be reached in the management of patients requesting assistance for OM issues. The study has, however, different limitations. First, the relatively small sample size of the group, which was influenced by the COVID pandemic that limited the access of patients to hospital facilities. Second, the method of retrospective chart review that does not allow to analyse other factors in the referral. Third, this is a single-center study; therefore, the results are not generalizable on a large-scale population.

## 5. Conclusions

This study analyzes the referral pattern to OM in Italy, highlighting the growing demand for assistance in this field related to the high incidence of oral diseases and the crucial role that OM services play. The results of this study demonstrate a low grade of correct diagnosis by referral provider with the consequent need to implement curricula among dentistry and medical students. Finally, it is imperative that OM becomes even more of a reference for the medical community as well, in order to provide a complete medical assistance to patients.

## Figures and Tables

**Table 1:** The demographic characteristic of patients.

	N	(%)
**All patients**	583	
**Age, mean years ± SD**	56.6 ± 16.2	
**Gender**		
Male	218	37.4
Female	365	62.6
**Risk factor**		
Smoking status		
Never	308	52.8
<5	120	20.6
5–15	113	19.4
>15	42	7.2
Alcohol use		
Never	215	36.9
Light drinker	219	37.6
Moderate drinker	127	21.8
Heavy drinker	22	3.8

The number of non-smokers were 308 (52.8%), whereas the number of smokers was 275 (47.2%). Regarding alcohol consumption, 215 (36.9%) patients refrained completely from alcohol consumption, and 368 (63.1%) reported consuming alcohol.

**Table 2:** Diagnostic tests ordered by referring providers.

Diagnostic Test	N
Blood examination	80
Biopsy	56
Orthopantomogram	53
Lingual swab	42
Computed tomography maxillo-facial	26
Gastroscopy	10
Maxillo-facial MRI	8
Brain MRI	6
General urine test	2
Others	25

**Table 3:** Reasons for referral.

Disease	N	(%)
*Immune-mediated mucosal conditions*	231	39.6
Oral lichen planus	164	28.1
Orofacial granulomatosis	29	5
Recurrent aphthous	19	3.3
Geographic tongue	18	3.1
Pemphigus disease	13	2.2
Plasma cell mucositis	6	1
Pemphigoid disease	4	0.7
Erythema multiforme	3	0.5
Behcet’s disease	1	0.2
*Orofacial pain disorders*	147	25.2
Burning mouth syndrome	111	19
Persistent idiopathic facial pain	30	5.1
Trigeminal neuralgia	4	0.7
Myofascial pain	1	0.2
Globus pharyngeus	1	0.2
*Benign tumors or neoplasms*	32	5.5
Fibroma	20	3.4
HPV-related lesions	8	1.4
Peripheral giant cell granuloma	2	0.3
Lipoma	1	0.2
Varix	1	0.2
*Dysplasia and cancerous conditions*	81	13.9
Leukoplakia	51	8.7
Oral carcinoma	27	4.6
Erythroplakia	3	0.5
*Reactive keratosis*	12	2
*Salivary gland disorders*	11	1.9
Sjogren’s syndrome	6	1
Mucocele	3	0.5
Sialadenitis	5	0.9
*Infections (Oral candidiasis)*	15	2.6
*Osteonecrosis of the jaws*	13	2.2
*Other mucosal and gingival lesions*	22	3.8
*Others* *	16	2.7

* Other conditions not included in the previous categories (dental pathology, normal variants, no disease).

## Data Availability

The data presented in this study are available on request from the corresponding author.
